# Retrospective Audit on Liaison Psychiatry Referrals

**DOI:** 10.1192/bjo.2026.11703

**Published:** 2026-06-30

**Authors:** Denise Hencil, Carlos Sánchez Belmar, Catherine Corby

**Affiliations:** Health Service Executive, Limerick, Ireland

## Abstract

**Aims:**

This audit compares the old and new referral forms to determine whether the redesigned version captures the required data more effectively, whether certain fields are unnecessary, and whether further improvements are needed. It also explores whether the older form encouraged more information or whether both forms still fall short in key areas.

**Methods:**

Data were obtained from stored copies of liaison psychiatry referrals submitted to University hospital of Limerick. A total of 100 referrals were reviewed. Two samples were created:

Baseline audit included 50 referrals randomly selected from January to April 2024 (using the old referral form)

Re Audit 50 included referrals randomly selected from January to April 2025 (using the redesigned referral form)

Data were collected and analysed. Each referral was reviewed against the predefined checklist of required data items derived from the 2025 redesigned referral form.

A structured Excel spreadsheet was used to record the presence or absence of each criterion for all referrals. Analysis was performed to compare completion rates between the old and new forms, assess compliance with the working standard, and identify areas requiring further refinement.

**Results:**

Overall, the redesigned referral form demonstrated significant improvement in the completeness and consistency of information provided across nearly all domains.

The new referral form showed compliance for patient identifiers and referrer details. In contrast, the old form showed inconsistent completion rates for key administrative data, particularly GP details, referring doctor, referrer contact number, and email.

Medical status, suspected psychiatric symptoms, duration, and pattern of symptoms were better captured in the new form compared to the old form.

Cognitive screening items such as alertness, AMT4 score, and counting months backwards were rarely recorded in the old referrals but were captured with much higher consistency in 2025.

The reason for admission was well documented in both forms, though more consistently in the new version.

Documentation of previous psychiatric diagnoses, current psychiatric medications, and attempts to treat prior to referral improved markedly with the new form. These elements were commonly missing or incompletely described in the old referrals.

Risk documentation was among the most significant improvements

**Conclusion:**

The redesigned 2025 referral form clearly improved the completeness of information submitted to liaison psychiatry, as demonstrated by the higher documentation rates across nearly all criteria. However, despite these improvements, several challenges remain that impact the practical usability and clinical value of the referral information received. This audit will inform a new service and quality development project.

